# Risk-adapted venous thromboembolism prophylaxis in Asian patients admitted to medical intensive care unit: a prospective controlled trial

**DOI:** 10.1186/s12959-025-00793-x

**Published:** 2025-11-04

**Authors:** Peampost Sinsakolwat, Konlawij Trongtrakul, Pattraporn Tajarernmuang, Nakarin Inmutto, Piangrawee Niprapan, Lalita Norasetthada, Adisak Tantiworawit, Ekarat Rattarittamrong, Thanawat Rattanathammethee, Sasinee Hantrakool, Pokpong Piriyakhuntorn, Nonthakorn Hantrakun, Teerachat Punnachet, Sirichai Srichairatanakool, Chatree Chai-Adisaksopha

**Affiliations:** 1 Division of Hematology, Department of Internal Medicine, Faculty of Medicine, Chiang Mai University, Chiang Mai, 50200 Thailand; 2 Division of Pulmonary and Critical Care Medicine, Department of Internal Medicine, Faculty of Medicine, Chiang Mai University, Chiang Mai, Thailand; 3 Division of Diagnostic radiology, Department of Radiology, Faculty of Medicine, Chiang Mai University, Chiang Mai, Thailand

**Keywords:** VTE prophylaxis, Caprini score, HAS-BLED score

## Abstract

**Background:**

Critically ill patients are at high risk for venous thromboembolism (VTE). In non-Caucasian patients, routine thromboprophylaxis is controversial. No standard guidelines exist for critically ill Thai patients.

**Objectives:**

To evaluate the efficacy and safety of a risk-adapted VTE prophylaxis protocol in medically ill patients.

**Methods:**

A single-center, prospective pre- and post-implementation trial conducted from March to December 2024. Patients admitted to three medical ICUs were enrolled. Patients were stratified by risk of VTE and bleeding. In the pre-implementation phase, patients did not receive thromboprophylaxis, whereas in the post-implementation phase, patients received thromboprophylaxis with either pharmacological or mechanical prophylaxis. The primary outcome was the 45-day incidence of VTE, VTE-related death, and bleeding events. Secondary outcomes included all-cause mortality, ICU stay, and days on mechanical ventilation.

**Results:**

There were 462 patients enrolled with a mean age of 65.82 ± 16.65 years and 53.90% were male. In the post-implementation phase, 151 out of 211 patients (65.37%) received thromboprophylaxis, primarily with pneumatic compression (41.56%), low molecular weight heparin/unfractionated heparin (15.58%) and aspirin (7.36%). VTE events occurred in 14 patients (6.06%) in the pre-implementation group and 5 (2.16%) in the post-implementation group. The composite primary outcome occurred in 14 patients (6.06%, 95% CI 3.35–9.96%) in the pre-implementation group and 5 (2.16%, 95% CI 0.71–4.98%) in the post-implementation group (RR 0.35, 95% CI 0.13–0.97, *P* = 0.04). A competing-risks analysis showed that post-implementation group was associated with significantly lower risk of VTE (adjusted subdistribution hazard ratio 0.35, 95% CI 0.13–0.97; *p* = 0.043). No VTE-related deaths occurred. Overall bleeding occurred in 28.14% of pre-implementation and 32.03% of post-implementation patients (RR 1.13, 95% CI 0.86–1.50, *P* = 0.361). Major bleeding rates were 11.26% vs. 8.22%, respectively (RR 0.65, 95% CI 0.40–1.07, *P* = 0.075).

**Conclusion:**

A risk-adapted VTE prophylaxis protocol significantly reduced VTE incidence in critically ill Asian patients without increasing bleeding complication.

**Clinical trial registration:**

TCTR20230927002, First Posted Date: 27 September 2023.

**Supplementary Information:**

The online version contains supplementary material available at 10.1186/s12959-025-00793-x.

## Background

Critically ill medical patients are at high risk of venous thromboembolism (VTE) [1–3], including deep vein thrombosis (DVT) and pulmonary embolism (PE). The underlying factors contributing to VTE are often related to both acute and chronic illnesses. Additionally, medical interventions such as mechanical ventilation, sedation, vasopressor therapy, central venous catheterization, and other procedures may further increase the risk of VTE [4]. Patients with VTE have been reported to experience prolonged intensive care unit (ICU) stays, extended durations of mechanical ventilation, and an increased risk of in-hospital mortality [5]. Consequently, VTE in critically ill patients is associated with poorer outcomes [1, 4].

In Caucasian patients, several studies have demonstrated the efficacy of thromboprophylaxis. A network meta-analysis by Shannon M. et al. and the PROTECT trial indicated that prophylaxis with unfractionated heparin (UFH) or low molecular weight heparin (LMWH), compared to placebo, reduced the incidence of VTE without increasing the risk of major bleeding [6–8]. Additionally, data on aspirin from the ASPIRE and WARFASA trials showed that aspirin reduced the risk of VTE recurrence and major adverse cardiac events [9–11]. Rivaroxaban and apixaban was non-inferior to LMWH, though the bleeding risk remained uncertain [12–14]. In patients with a clear contraindication to anticoagulation, mechanical prophylaxis, such as intermittent pneumatic compression (IPC) devices, was preferred for VTE prophylaxis. 

However, the incidence of VTE in medically ill patients was lower among the Asian population, ranging from 7 to 15.8% [1, 15]. Therefore, thromboprophylaxis remains controversial in Asian patients who are admitted to medical ICU. This study aimed to evaluate the efficacy and safety of a risk-adapted VTE prophylaxis protocol in Asian patients who were admitted to the medical ICU.

## Methods

### Study design

A single-center, prospective pre- and post- implementation trial was conducted across three medical ICUs in a tertiary care center in Thailand from March 2024 to December 2024. The inclusion criteria comprised patients admitted to the medical ICU who were older than 18 years, had an expected ICU stay of more than 72 h, and met at least one of the following conditions: acute respiratory failure requiring invasive mechanical ventilation, acute kidney injury requiring renal replacement therapy (RRT), sepsis as defined by the Third International Consensus Definitions for Sepsis and Septic Shock [16], or hemodynamic instability, such as systolic blood pressure < 90 mmHg or a drop > 40 mmHg, mean arterial pressure < 65 mmHg, oliguria (< 0.5 mL/kg/hour), and capillary refill time > 4.5 s or cool extremities [17]. Patients receiving anticoagulants, those with known thrombophilia, those with active VTE or those who had major surgery within three months before recruitment were excluded. This study was approved by the Research Ethics Committee, Study Number MED-2566-0403 (approval number 073/2024). Written informed consent was obtained from all patients or their relatives. The trial was registered in Clinical Trial Registration TCTR20230927002, First Posted Date :27 September 2023.

### Study procedures

The risk assessment was conducted upon patient admission using the Caprini score [18] and HAS-BLED score [19]. The Caprini score stratified VTE risk, with a cutoff of 3—scores ≥ 3 was defined as high risk, while < 3 was low risk. While, the HAS-BLED score estimated major bleeding risk, with the cutoff—3 or higher indicating high risk and below 3 indicating low risk [18, 19].

The study was conducted in two phases. In the pre-implementation phase, patients received standard care, which included early ambulation and VTE surveillance. In the post-implementation phase, a risk-adapted VTE prophylaxis strategy was introduced based on the risk of VTE and bleeding complications. Patients at high risk of VTE with a low risk of bleeding were recommended to receive pharmacologic prophylaxis, including anticoagulants or aspirin at the discretion of the attending physician. We decided to include aspirin based on its demonstrated efficacy in VTE prevention among medical patients [9–11, 20]. Anticoagulation was recommended as the first-line option for patients with an indication for pharmacologic prophylaxis. However, in circumstances where anticoagulation was not deemed the most appropriate option, aspirin was recommended as the alternative. Those at high risk of both VTE and bleeding were advised to use mechanical prophylaxis—Intermittent pneumatic compression (IPC; LGT-2201DVT^®^, Guangzhou Longest Medical Technology Co., Ltd, China). Patients at low risk of VTE, regardless of bleeding risk, were monitored with surveillance. VTE prophylaxis was discontinued if contraindicated or if patients experienced major complications of either pharmacological or mechanical prophylaxis. VTE prophylaxis was administered at the time of admission and continued until the patient was discharged from the ICU. Details regarding the type of prophylaxis used in the risk-adapted VTE prophylaxis protocol was presented in Supplementary Fig. 1.

Patients were screened for proximal DVT using Doppler ultrasound within 72 h of ICU admission. Serial Doppler ultrasounds were conducted on day 7 and then weekly (or earlier if clinically indicated) until ICU discharge. Additionally, signs and symptoms of PE were monitored as part of routine care by attending doctors. If clinical suspicion arose, pulmonary angiography computed tomography was performed to confirm the diagnosis.

The Doppler ultrasound screening was performed by trained ICU physicians. The three-point compression ultrasound protocol involved screening the common femoral vein at the inguinal crease, the superficial femoral vein above the adductor canal, and the popliteal vein in the popliteal fossa. A positive DVT screening was determined if the veins were noncompressible or if an intraluminal thrombus was directly visualized. After recording the results, patients were referred to the radiology department for a whole-leg compression ultrasound confirmed by a radiologist blinded to their participation in this study. Patients were followed for up to 45 days following discharge from the critical care unit.

### Data collection

Demographic data were collected including age, sex, comorbidities, admission diagnosis, and severity of illness based on the APACHE II score [21]. Caprini and HAS-BLED scores were evaluated at the time of admission [19]. Treatments and interventions during the patient’s hospital stay, such as RRT, mechanical ventilation, vasopressor, neuromuscular blocking agents, central venous catheter, and type of thromboprophylaxis were collected. Patient outcomes consisted of VTE or VTE-related death, bleeding event, duration of mechanical ventilation, all-cause mortality, and length of hospital stay.

The primary outcomes were the incidence of VTE, VTE-related death, and overall bleeding, major bleeding, and non-major bleeding, as defined by the International Society on Thrombosis and Haemostasis (ISTH) criteria [22], during ICU stay and up to 45 days after ICU discharge. Secondary outcomes included all-cause mortality, ICU length of stay, and number of days on mechanical ventilator.

### Statistical analysis

Demographic data were presented as descriptive statistics. Categorical variables were reported as counts and percentages, while continuous variables were presented as means and standard deviations (SD). Patient characteristics in the pre- and post-implementation phases were compared using the independent t-test for continuous variables and the Chi-square or Fisher’s exact test for categorical variables.

VTE incidence was reported as percentages with 95% confidence intervals (CIs). A log-binomial regression model was used to estimate the risk ratio (RR) and 95% CI of the risk factor. We performed a competing-risks analysis of cumulative incidence, treating deaths prior to VTE as competing events. Subdistribution hazard ratios (SHR) were estimated using the Fine–Gray model. We performed a multivariable Cox proportional hazards model to adjust for potential confounders. A p-value of < 0.05 was considered statistically significant. All analyses were performed using Stata version 17 (StataCorp).

The sample size calculation was based on the estimated incidence of proximal DVT in medically ill patients who did not receive thromboprophylaxis was 15.8% [15]. Assuming the incidence of DVT in the thromboprophylaxis group would be 7.5%, the required sample size was 462 patients, with 231 in each phase.

## Results

Between March and December 2024, 527 patients were admitted to the medical ICU. Of these, 262 patients in the pre-implementation phase and 265 in the post-implementation phase underwent screening for trial eligibility. A total of 31 patients in the pre-implementation phase and 34 in the post-implementation phase were excluded due to not meeting inclusion criteria or declining participation. A total of 462 patients were enrolled in the study, with 231 in each phase, as shown in Supplementary Fig. 2.

Baseline demographics and clinical characteristics are summarized in Table 1. The mean (SD) age was 65.82 (16.65) years, and 53.90% were male. No significant differences were found in comorbidities, initial laboratory values, or treatments during ICU stay. Nearly 97% of patients required mechanical ventilation and about half needed vasopressors. The leading cause of ICU admission was acute pneumonia (53.25%), followed by septic shock (47.62%), acute decompensated heart failure (9.09%), urinary tract infection (6.71%), and COPD exacerbation (7.14%). The etiologies were largely similar across the phases, except for a higher incidence of acute decompensated heart failure in the post-implementation phase and a higher number of septic shock cases in the pre-implementation phase. Mortality prediction score (APACHE II) was comparably high across both phases. Most patients had a high Caprini score (436 of 462, 94.12%) and a low HAS-BLED score (396 of 462, 85.71%), with consistent trends across phases.

**Table 1:** Baseline characteristics of the patients in the pre-implementation and post-implementation phases

Characteristics	Pre-implementation phase (*n* = 231)	Post-implementation phase (*n* = 231)	*P* value
Age years (mean ± SD)	66.51 ± 16.28	65.13 ± 17.02	0.374
Male, n (%)	128 (55.41)	121 (52.38)	0.514
Malignancy, n (%)	65 (28.14)	55 (23.81)	0.289
Diabetes, n (%)	55 (25.7)	62 (26.83)	0.395
Congestive heart failure, n (%)	17 (7.42)	13 (5.63)	0.435
Hypertension, n (%)	100 (43.67)	103 (44.59)	0.842
Hyperlipidemia, n (%)	63 (27.51)	54 (23.38)	0.309
Ischemic heart disease, n (%)	17 (7.36)	18 (7.79)	0.860
Stroke/old CVA, n (%)	26 (11.26)	24 (10.39)	0.765
Vascular disease(PAD), n (%)	10 (4.37)	11 (4.76)	0.839
Chronic kidney disease, n (%)	34 (14.85)	36 (15.58)	0.826
Arrhythmia, n (%)	18 (7.79)	15 (6.49)	0.588
Asthma/COPD, n (%)	22 (9.52)	23 (9.96)	0.875
Cirrhosis, n (%)	17 (7.36)	10 (4.33)	0.165
Previous VTE, n (%)	5 (2.16)	1 (0.43)	0.100
Etiologies of admission			
Septic shock, n (%)	121 (52.38)	99 (42.86)	0.040
Pneumonia, n (%)	123 (53.25)	123 (53.25)	1.000
ARDS, n (%)	11 (4.76)	19 (8.23)	0.131
AE-COPD/Asthma, n (%)	17 (7.36)	16 (6.93)	0.857
Seizure, Status epilepticus, n (%)	8 (3.46)	12 (5.19)	0.360
Acute decompensated heart failure, n (%)	8 (3.46)	34 (14.72)	< 0.001
Acute liver failure/decompensation, n (%)	8 (3.46)	6 (2.60)	0.587
UGIH/LGIH, n (%)	8 (3.46)	6 (2.60)	0.587
AKI with RRT, n (%)	9 (3.90)	9 (3.90)	1.000
DKA/HHS, n (%)	7 (3.03)	10 (4.33)	0.458
Urinary tract infection, n (%)	19 (8.23)	12 (5.19)	0.297
Skin and soft tissue infection, n (%)	2 (0.87)	0 (0.00)	0.156
Febrile neutropenia, n (%)	3 (1.30)	0 (0.00)	0.082
Enterocolitis, n (%)	9 (3.90)	5 (2.16)	0.278
Post cardiac arrest, n (%)	13 (5.63)	9 (3.90)	0.382
Other, n (%)	46 (19.91)	31 (13.42)	0.061
Caprini score			
Low (< 3), n (%)	17 (7.36)	9 (3.90)	0.106
High (> = 3), n (%)	214 (92.64)	222 (96.10)	
HAS-BLED score			
Low (< 3), n (%)	198 (85.71)	198 (85.71)	1.000
High (> = 3), n (%)	33 (14.29)	33 (14.29)	
ICU acquired risk factors			
Mechanical ventilator, n (%)	225 (97.40)	220 (95.24)	0.217
Renal replacement therapy, n (%)	44 (19.05)	29 (12.55)	0.056
Vasopressor, n (%)	123 (53.25)	119 (51.52)	0.709
Neuromuscular blocking agent, n (%)	9 (3.90)	11 (4.76)	0.648
Central line, n (%)	70 (30.30)	81 (35.06)	0.275
APACHE-II score (mean ± SD)	22.59 ± 6.69	21.35± 6.86	0.065
Index Hospitalization Characteristics			
Hb, g/dL (mean ± SD)	9.88 ± 2.61	10.27 ± 2.84	0.127
WBC, x10^9^/L; median (IQR1-IQR3)	11.38 (7.70–16.11.70.11)	10.80 (7.26–15.39)	0.265
Platelet, x10^9^/L; median (IQR1-IQR3)	221(121–309)	215(117–313)	0.777
Creatinine mg/dL; median (IQR1-IQR3)	1.11 (0.68–2.09)	1.01 (0.67–1.87)	0.743
GFR mL/min/1.73m^2^; median (IQR1-IQR3)	44.86 (24.08–73.97)	47.22 (26.71–83.03)	0.656
Number of ultrasound; median (IQR1-IQR3)	1 (1–2)	2 (1–3)	0.167
Mortality in 45 days, n (%)	92 (39.83)	49 (21.21)	< 0.001
Ventilator days; median (IQR1-IQR3)	5 (2–9)	4 (2–10)	0.834
ICU stay; median (IQR1-IQR3)	7 (4–13)	7 (4–12)	0.953

*Abbreviation*: *CVA* Cerebrovascular disease, *PAD *Peripheral artery disease, *COPD* Chronic obstructive pulmonary disease, *VTE* Venous thromboembolism, *ARDS* Acute respiratory distress syndrome, *AE-COPD*,Acute exacerbations of chronic obstructive pulmonary disease, *UGIH* Upper gastrointestinal hemorrhage, *LGIH *Lower gastrointestinal hemorrhage, *AKI* Acute kidney injury, *RRT *Renal replacement therapy, *DKA* Diabetic ketoacidosis, *HHS* Hyperosmolar hyperglycemic state, *APACHE* Acute Physiology and Chronic Health Evaluation, *Hb* Hemoglobin, *WBC* white blood cell, *GFR* Glomerular filtration rate, *ICU* intensive care unit, *IQR* interquatile range

### Thromboprophylaxis

Supplementary Table 1 demonstrates the type of thromboprophylaxis in patients in two phases. During the pre-implementation phase, nearly all patients (99.13%) did not receive prophylaxis. In the post-implementation phase, 65.37% received thromboprophylaxis. The majority used intermittent pneumatic compression (41.56%), followed by pharmacologic prophylaxis with low molecular weight heparin (LMWH)/Unfractionated heparin (UFH) (15.58%) and aspirin (7.36%). Of these, 7 patients received UFH. A small number of patients received direct oral anticoagulants (DOACs) or warfarin. None of the patients receiving mechanical prophylaxis discontinued treatment. However, five patients who developed major bleeding while on antithrombotic therapy discontinued prophylaxis.

### Primary outcomes

**Fig. 1:**
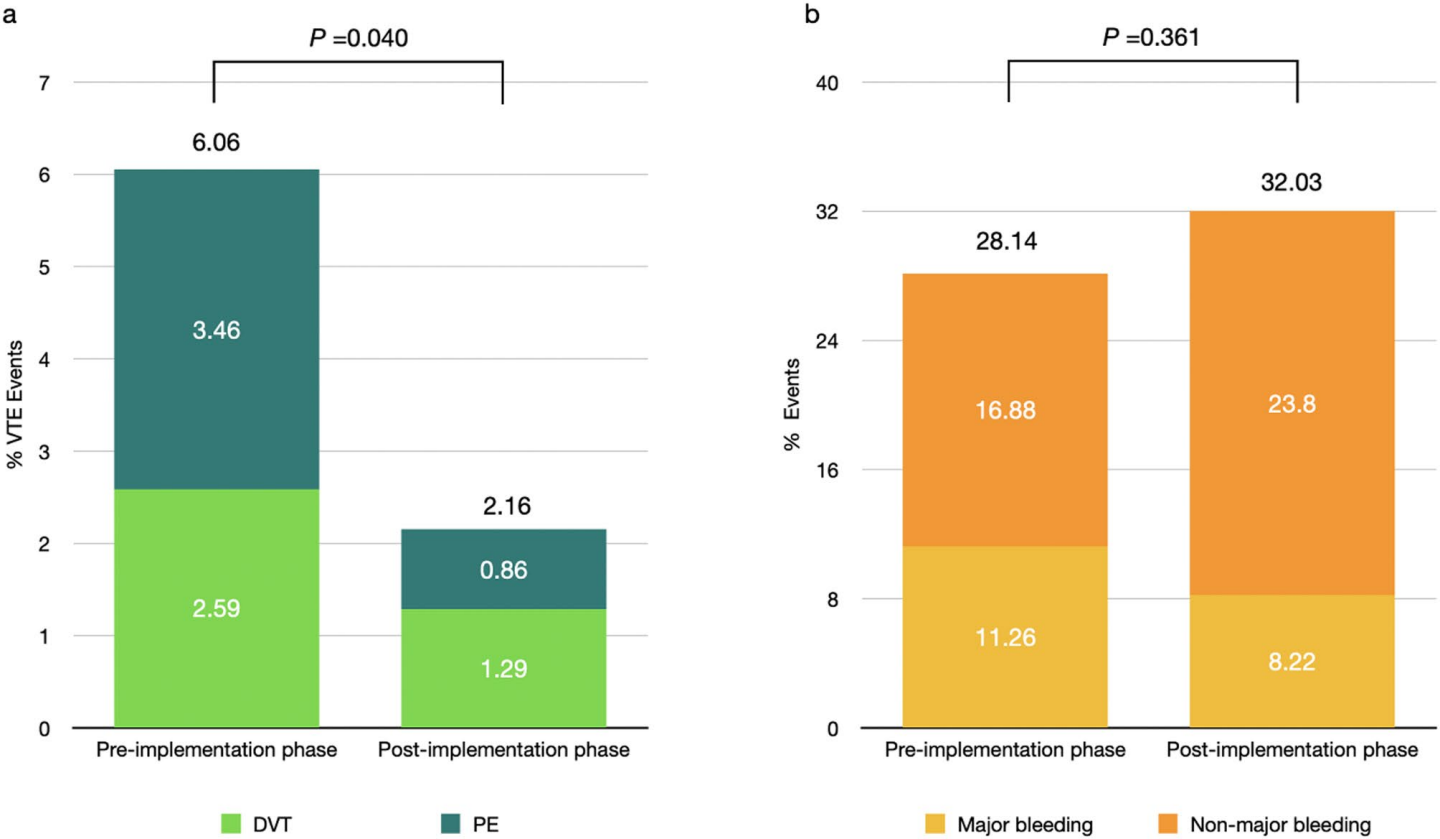
**A** The bar chart showed the incidence of VTE, DVT, and PE between pre-implementation and post-implementation phase, **B** showed the number of overall bleeding, major bleeding and minor bleeding in patients between pre-implementation and post-implementation phase

Figure 1A demonstrates the VTE event in patients in the pre- and post-implementation phases. VTE events occurred in 6.06% (95% CI 3.35–9.96%) of patients in the pre-implementation phase and 2.16% (95% CI 0.71–4.98%) in post-implementation. Risk-adapted thromboprophylaxis significantly reduced the risk of VTE, RR 0.35 (95%CI 0.13–0.97, *P* = 0.04). In the competing-risks analysis, risk-adapted thromboprophylaxis significantly reduced the risk of VTE (SHR 0.35, 95% CI 0.13–0.97, *p* = 0.043). After adjustment for age, sex, and underlying cancer, the adjusted SHR for VTE was 0.35 (95% CI 0.13–0.97, *p* = 0.043), consistent with the unadjusted analysis. Patients with cancer were associated with increased risk of VTE (SHR 4.69, 95% CI 1.91–11.47, *P* = 0.001). The absolute risk reduction (ARR) was 3.93%, and the number needed to treat (NNT) was 26. Most of the patients (80%) who developed VTE in the post-implementation period did not receive thromboprophylaxis (Supplementary Table 2). There was no VTE-related death in both phases.

The median time to DVT diagnosis was 11 days (range: 3–45 days, IQR1-IQR3: 8–22 days), while the median time to PE diagnosis was 6 days (range: 3–45 days, IQR1-IQR3: 4–11 days). Seven out of nine DVT patients were asymptomatic, with DVT detected through ultrasound screening and confirmed by a radiologist. In contrast, seven out of ten PE patients were symptomatic and underwent pulmonary angiography computed tomography for diagnosis. The remaining three were incidentally diagnosed with PE during computed tomography performed for other indications, such as suspected complicated pneumonia or vascular injury.

Figure 1B and Table 2 demonstrates bleeding events. Overall bleeding events were 28.14% in pre-implementation and 32.03% after implementation. Risk-adapted thromboprophylaxis did not significantly increase the risk of overall bleeding in the post-implementation phase as compared to the pre-implementation phase (RR 1.13, 95% CI 0.86–1.50, *P* = 0.36). Major bleeding was slightly higher in the pre-implementation phase, (11.26% vs. 8.22%, RR = 0.64, 95%CI 0.39–1.04 *P* = 0.075). Non-major bleeding showed a slightly increase in the post-implementation phase (16.88% vs. 23.80%, RR = 1.23, 95% CI 0.97–1.57 *P* = 0.080). Table 2 demonstrates the site of bleeding in two phases. Gastrointestinal bleeding was the most common site of bleeding, accounting for more than half of all bleeding cases **(**Table 2**)**. No complications related to mechanical prophylaxis were reported.

**Table 2:** Bleeding events in the pre-implementation and post-implementation phases

Events, *n* (%)	Pre-implementation phase (*n* = 231)	Post-implementation phase (*n* = 231)	RR	95% CI	*P* value
Overall bleeding	65 (28.14)	74 (32.03)	1.13	0.86–1.50	0.361
Major bleeding	26 (11.26)	19 (8.22)	0.64	0.39–1.04	0.075
UGIH	17 (7.36)	9 (4.29)	0.72	0.41–1.25	0.227
LGIH	5 (2.16)	6 (2.59)	1.64	0.58–4.59	0.345
Hematuria	1 (0.43)	3 (1.29)	4.10	0.46–36.47	0.205
Hemoptysis	2 (0.87)	4 (1.73)	2.73	0.55–13.43	0.215
Soft tissue bleeding	1 (0.43)	2 (0.86)	2.73	0.26–28.02	0.375
Others^a^	2 (0.87)	2 (0.87)	1.36	0.21–8.86	0.741
Non-major bleeding	39 (16.88)	55 (23.80)	1.23	0.97–1.57	0.080
UGIH	32 (13.85)	38 (16.45)	0.84	0.66–1.05	0.143
LGIH	2 (0.87)	6 (2.59)	2.12	0.45–9.99	0.339
Hematuria	3 (1.30)	6 (2.59)	1.65	0.45–6.00	0.444
Hemoptysis	5 (2.16)	5 (2.16)	0.709	0.22–2.28	0.563
Soft tissue bleeding	0 (0.00)	2 (0.86)	NA	NA	0.229
Others^a^	0 (0.00)	7 (3.03)	NA	NA	0.021
Mechanical prophylaxis related complication	0 (0.00)	0 (0)	NA	NA	NA

*Abreviation:* *UGIH* Upper gastrointestinal hemorrhage, *LGIH* Lower gastrointestinal hemorrhage, *NA* not applicable, *RR* relative risk, *95% CI* 95% confidence interval

^a^Others including hemoperitoneum, diffuse alveolar hemorrhage, epistaxis, bleeding from pressure ulcer, tracheostomy site bleeding, double lumen catheter site bleeding

### Secondary outcomes

There was no significant difference in the median ICU length of stay (7 days; range, 2–86 days vs. 7 days; range, 2–73 days, *P* = 0.953) and ventilator days (5 days; range, 1–33 days vs. 4 days; range, 1–42 days, *P* = 0.834) between pre- and post-implementation phases, respectively. The 45-day mortality rate was higher in the pre-implementation phase than in the post-implementation phase (39.83% vs. 21.21%, *P* < 0.001). Besides, patients with VTE were associated with an increased risk of 45-day mortality as compared to those without VTE (RR, 3.31; 95% CI, 1.30–8.41; *P* = 0.012).

## Discussion

This study evaluated the incidence of VTE, DVT, and acute PE, in Asian patients admitted to the medical ICU. Our findings demonstrate that implementing a risk-adapted thromboprophylaxis protocol significantly reduced VTE incidence from 6.06% to 2.16%. These results highlighted the potential benefit of individualized prophylaxis in critically ill medical patients.

Baseline characteristics were generally comparable across phases. However, the non-randomized design may have introduced imbalances, particularly in admission etiologies: septic shock was more frequent in the pre-implementation phase, whereas acute decompensated heart failure was predominated in post-implementation. Although definitive evidence was lacking regarding which condition conferred greater risk of VTE or bleeding, both were associated with increased risk [23, 24]. Further studies were needed to clarify the impact of admission etiologies on these outcomes.

It was well established that VTE incidence in Asian populations was lower than in Caucasians. The incidence of VTE in critically ill Caucasian patients was 28–31% without thromboprophylaxis and 5.4–9.6% with LMWH or heparin [3, 6, 25, 26]. The incidence of VTE in medically ill Thai patients who did not receive thromboprophylaxis in this present study was consistent with previous studies, reporting rates between 7% and 15.8% [1, 4, 15]. To date, there have been no reports on prophylaxis utilization in Thailand. This present study demonstrated an approximately 60% reduction of the risk of VTE in critically ill patients who received risk-adapted thromboprophylaxis.

Several studies have highlighted the negative impact of VTE, as all-cause mortality increases in patients who develop VTE during ICU admission [5, 27, 28]. The mortality rate was 11.4% in patients with asymptomatic VTE (HR, 2.31; 95% CI, 1.52–3.51; *P* < 0.0001) and 29.2% in those with symptomatic VTE (HR, 9.42; 95% CI, 4.18–21.20; *P* < 0.0001), compared to patients without VTE [29]. Our study demonstrated a similar association with increased 45-day mortality in VTE patients (RR, 3.31; 95% CI, 1.30–8.41; *P* = 0.012).

In this present study, most DVT cases occurred during the third to fourth screening, approximately 10–14 days after admission, while PE tended to occur earlier, around 6 days after admission. Our results showed a later occurrence compared to other studies, where DVT typically arose within 48 h and peaked by day 7 of admission [1, 15, 30]. Nearly all events occurred during hospitalization, with only one patient in this present study experiencing a VTE event after hospital discharge. This aligns with the known risk period for VTE, which extends up to 30–45 days after discharge [31].

Previous studies indicated that thromboprophylaxis shortened ICU stays and reduced hospital mortality. These studies showed higher ICU and hospital mortality rates in patients who did not receive thromboprophylaxis within 24 h of ICU admission compared to those treated early (ICU mortality: 7.6% vs. 6.3%, *P* = 0.001; hospital mortality: 11.2% vs. 10.6%, *P* = 0.003) [5, 27, 32]. This present study’s findings aligned with these, as the 45-day follow-up mortality rate was higher in the pre-implementation phase, suggesting the benefit of early thromboprophylaxis.

Several risk-assessment models have been developed for medical inpatients to guide individualized VTE prophylaxis. The American Society of Hematology (ASH) guidelines recommended the Padua and IMPROVE scores, both externally validated with fair discrimination properties [8]. However, no standardized models existed for critically ill medical patients. The Caprini risk assessment score was initially developed and validated to predict postoperative VTE in surgical patients. Recent validation studies in medical patients have produced conflicting results. The Caprini score showed higher sensitivity than the Padua score, primarily due to its more comprehensive list of risk factors [18, 33]. The ASH guideline recommended the IMPROVE bleeding score [8], while the HAS-BLED score, validated in several studies, demonstrated strong predictive validity for bleeding risks in medical patients with VTE [19, 34]. Therefore, this study decided to adopt the Caprini and HAS-BLED scores, as supported by prior studies [19, 33].

As shown in Supplementary Table 1, the majority of patients in the pre-implementation phase (99.13%) did not receive thromboprophylaxis, whereas 34.63% of patients in the post-implementation phase did not receive prophylaxis. In Western countries, thromboprophylaxis is a standard practice, with reported utilization rates ranging from 33% to 100% among critically ill patients [35]. However, in Thailand there are currently no standardized national guidelines for critically ill patients. Consequently, decisions regarding the use of thromboprophylaxis and the choice of agent are determined by individual physician discretion. This reflects variability in physician practice patterns, particularly in the ICU setting, where some physicians remain cautious about pharmacologic prophylaxis despite guideline and protocol recommendations. We observed that IPC devices were the preferred choice among attending physicians, likely due to concerns about bleeding complications associated with pharmacologic prophylaxis.

Overall bleeding events in this study showed no significant difference between the pre- and post-implementation phases. Previous studies indicated that in patients at high VTE risk who received pharmacologic prophylaxis, bleeding incidence was comparable to those who did not [36]. Bleeding rates varied widely, ranging from 1.4% to 37.4%, likely due to differences in baseline risk factors and definitions of bleeding across studies [37–39]. Our study observed a relatively high bleeding rate (30.08%), possibly due to several predisposing factors for bleeding such as low platelet count, prolonged activated partial thromboplastin time (aPTT), and a high number of patients receiving RRT [40]. Gastrointestinal hemorrhage constituted the majority of bleeding events in this study. This aligned with several reviews indicating that, without stress ulcer prophylaxis, clinically important gastrointestinal bleeding occurs in 4.2% to 25% of critically ill patients [41, 42]. No complications were observed with the use of IPC device, supporting its safety as demonstrated in prior studies [43]. These findings reassured that the implementation of thromboprophylaxis did not increase the risk of bleeding complications.

The strengths of the present study included a large sample size with adequate statistical power, a prospective cohort design, and the consecutive enrolment of a broad population of critically ill patients. Duplex ultrasonography was performed by trained ICU physicians and subsequently reviewed and confirmed by a radiologist ensuring diagnostic accuracy. Lastly, all patients were followed for up to 45 days, enabling the detection of VTE event that might occur after ICU discharge [31].

The limitations of our trial included the fact that VTE and bleeding risk scores were calculated on the first day of admission. This timing may have resulted in the omission of factors that occurred after admission, potentially introducing immortal time bias and underestimating the Caprini and HAS-BLED scores. Furthermore, treatment physicians were likely to choose mechanical over pharmacological prophylaxis possibly due to their concerns regarding bleeding events, despite pharmacological prophylaxis being the recommended first-line approach according to standard guidelines [7, 8]. Lastly, data on fatalities related to bleeding were not initially planned to be collected. Moreover, since the causes of death in critically ill medical patients are often multifactorial, it is difficult to clearly identify the primary cause of death.

## Conclusion

The incidence of VTE among medically critically ill Asian patients was substantially high. Risk-adapted thromboprophylaxis, based on the Caprini and HAS-BLED scores, demonstrated both efficacy and safety, effectively reducing the incidence of VTE without a corresponding increase in bleeding complications.

## Supplementary Information

Supplementary Material 1.

## Data Availability

The datasets used and/or analysed during the current study are available from the corresponding author on reasonable request.

## Abbreviations

VTE: Venous thromboembolism
ICU: Intensive care unit
DVT: Deep vein thrombosis
PE: Pulmonary embolism
UFH: Unfractionated heparin
LMWH: Low molecular weight heparin
IPC: Intermittent pneumatic compression
RRT: Renal replacement therapy
DOACs: Direct oral anticoagulants
aPTT: Activated partial thromboplastin time
