# Supplementary material for: Risk-adapted venous thromboembolism prophylaxis in Asian patients admitted to medical intensive care unit: a prospective controlled trial
Source: Thromb J. 2025 Nov 4;23:106. doi: 10.1186/s12959-025-00793-x (PMC12584491; doi:10.1186/s12959-025-00793-x)
Supplement: Supplementary file 1 — Supplementary Material 1. [file 12959_2025_793_MOESM1_ESM.docx]

**Supplementary data**

**Supplementary Table 1** The prophylactic measures determined by the attending physicians for the pre-implementation and post-implementation phases.

| **Prophylaxis** | **Total (N=462)** | **Pre-implementation phase (n=231)** | **Post-implementation phase (n=231)** | ***P* value** |
| --- | --- | --- | --- | --- |
| **None, n (%)** | 309 (66.88) | 229 (99.13) | 80 (34.63) | <0.001 |
| **Mechanical prophylaxis** |  |  |  |  |
| Intermittent pneumatic compression, n (%) | 97 (21.00) | 1 (0.43) | 96 (41.56) | <0.001 |
| **Pharmacological prophylaxis** |  |  |  |  |
| LMWH/UFH, n (%) | 37 (8.00) | 1 (0.43) | 36 (15.58) | <0.001 |
| DOAC, n (%) | 2 (0.44) | 0 (0.00) | 2 (0.86) | 0.498 |
| Aspirin, n (%) | 17 (3.68) | 0 (0.00) | 17 (7.36) | 0.000 |
| Warfarin, n (%) | 1 (0.22) | 0 (0.00) | 1 (0.43) | 0.317 |

Abreviation : LMWH, low molecular weight heparin; UFH, unfractionated heparin; DOAC; direct oral anticoagulant

**Supplementary Table 2** Venous thromboembolism (VTE) events and thromboprophylaxis in the pre-implementation and post-implementation phases

| **Prophylaxis** | **Total VTE (N=19)** | **VTE in Pre-implementation phase (n=14)** | **VTE in Post-implementation phase (n=5)** |
| --- | --- | --- | --- |
| **None, n (%)** | 18 (94.73) | 14 (100) | 4 (80) |
| **Mechanical prophylaxis** |  |  |  |
| Intermittent pneumatic compression, n (%) | 0 (0) | 0 (0) | 0 (0) |
| **Pharmacological prophylaxis** |  |  |  |
| LMWH/UFH, n (%) | 1 (5.26) | 0 (0) | 1 (20) |
| DOAC, n (%) | 0 (0) | 0 (0) | 0 (0) |
| Aspirin, n (%) | 0 (0) | 0 (0) | 0 (0) |
| Warfarin, n (%) | 0 (0) | 0 (0) | 0 (0) |

**Supplementary Table 3** Major and minor bleeding rates stratified by prophylaxis type

| **Bleeding events, n(%)**  **Prophylaxis** | **Major bleeding** | **Minor bleeding** |
| --- | --- | --- |
| **Intermittent pneumatic compression (n=97)** | 9 (9.27) | 28 (28.86) |
| **LMWH/UFH (n=37)** | 4 (10.81) | 10 (27.02) |
| **Aspirin (n=17)** | 1 (5.88) | 5 (29.41) |

**Supplementary Figure 1** Risk adapted VTE prophylaxis and dose of anticoagulant and antithrombotic drug in this present study

**
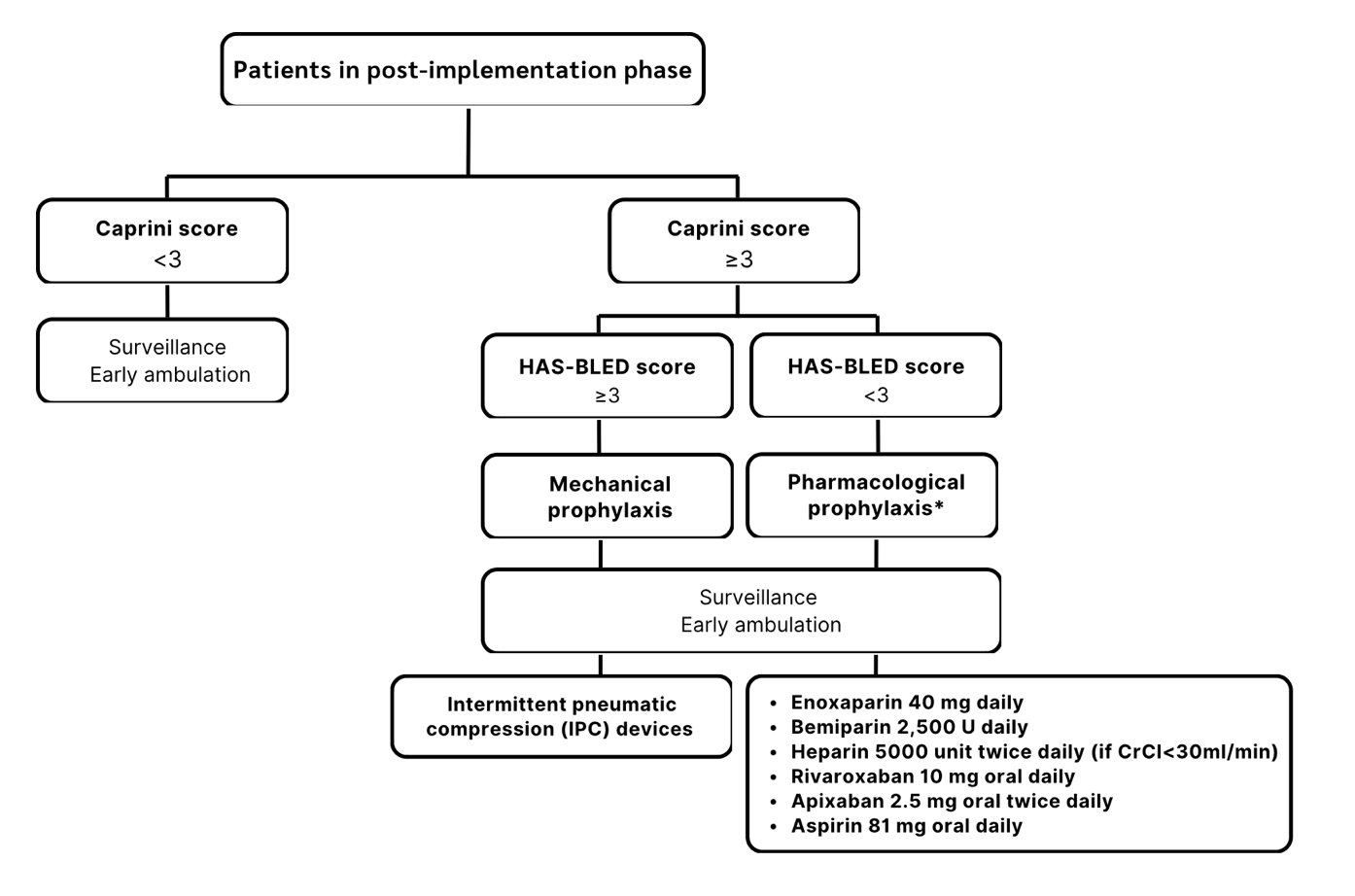
**

*This study recommended pharmacological prophylaxis as the first-line approach. However, attending physicians may choose either pharmacological or mechanical prophylaxis based on their preference and clinical judgment.

**Supplementary Figure 2** Consort diagram of the study

**
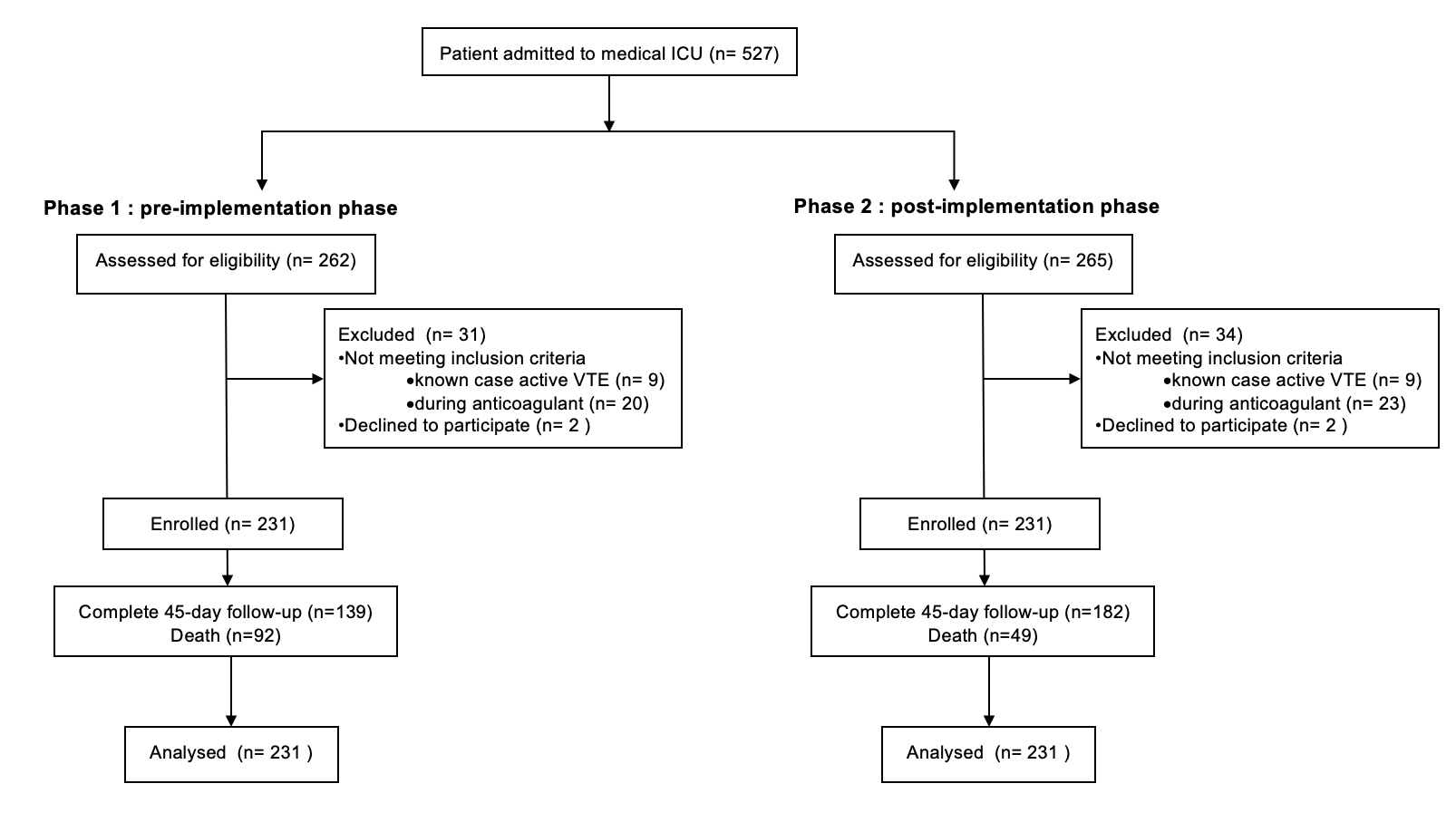
**
